# mSphere of Influence: Courage and Resilience in Life and Science

**DOI:** 10.1128/mSphere.00200-21

**Published:** 2021-03-17

**Authors:** Anat Florentin

**Affiliations:** a Department of Microbiology and Molecular Genetics, Faculty of Medicine, The Hebrew University of Jerusalem, Jerusalem, Israel

**Keywords:** microbiology, science history, career

## Abstract

Anat Florentin works in the field of molecular parasitology, studying the cell biology of malaria parasites. In this mSphere of Influence article, she reflects how the book *Brave Genius: a Scientist, a Philosopher, and Their Daring Adventures from the French Resistance to the Nobel Prize* by Sean B. Carroll (2013) made a powerful impact on her by telling scientific stories in the context of dramatic life events.

## COMMENTARY

How many of us are concerned about the role of science in our lives? I have often found that this path is far more than a professional endeavor. Even those who got here serendipitously, gradually embrace it as a way of life, affecting how we experience the world, interpret social events, and develop as individuals. I have spent too many hours thinking about these issues, in particular while I should have been doing productive benchwork or grant writing. One thing that consistently triggers these thoughts is reading about science and scientists. I find the concept of books as windows and mirrors extremely engaging (1), and in the context of science also very useful. A book can open a window and tell me stories I did not know, from perspectives others than mine. It can also be a mirror reflecting my own story in a way that makes me feel less alone.

There was a certain time in my life that the one book to rule them all was *Brave Genius* by Sean B. Carroll (2). If you haven’t read it, dear friends, please do. It is the 1940s and several French scientists have some serious problems. Not only that it is completely unclear how genes are regulated and what is the right way to go about it. It is also that, well, there are Nazis all over Paris. These are extremely dangerous times and future Nobel laurate Jacques Monod is joining the Resistance while the young Francois Jacob is fighting with the Free French forces in North Africa. It will be several horrific years before they will do their groundbreaking work at the Pasteur Institute and become the fathers of molecular biology. There is going to be some beautiful science emerging from the discovery of the lactose operon: new concepts such as gene regulation, enzyme induction, and bacterial metabolism to name a few. Just not *quite yet*. Right now, a few courageous men and women risk everything for what they believe is true and just.

A truly magnificent story, perfectly told by Prof. Carroll, a brave scientist himself. It had everything I could ask from a book—adventure and suspense; science and bigger-than-life scientists; and, as a cherry on top, a glimpse into the world of philosopher Albert Camus, another Resistance member and one of my all-time favorite writers. It can be easily understood how this novel opened a window showing how, during dark moments in human history, the deeds of these scientists kept the light on. But it was also encouraging on a more personal level, surprisingly becoming a mirror, and to understand that, I need to go a few years back in time.

I was starting my Ph.D. and was about to have my first baby. Hey, women have done it since the dawn of history, how hard can it be? Well, pretty darn hard, and shockingly awful at times. I was pregnant and, although I didn’t know it at the time, I had a syndrome called hyperemesis gravidarum. It would have helped to know that this debilitating state is actually a medical condition, that I am not weak or spoiled, and that all this self-blame is really unnecessary. Still, the hard truth was that every aspect of my life, my work included, was suffering. It seemed that my Ph.D. was not becoming as magnificent as I thought it should be, and that I was not scientist material after all.

Even if you find this logic flawed, this feeling might be familiar. We are all consumed by the myth of “The Scientist,” an individual who dedicates their life, without exception, to a brilliant career, and any deviation means mediocrity. And this is exactly why *Brave Genius* was so helpful: to start with, it pulled me out of my boring miserable life. This real-life drama, adventures, suspense, and the victory of the human spirit were all excellent antinausea medications, perfectly safe for use by sad, pregnant women. But the more I read, the more personal the story became, telling me that life gets in the way even for great scientists with huge intellects and enormous potential. Not your typical girl-power, feminist manifesto, but nevertheless it had the right impact. I wasn’t fighting Nazis, but I was fighting my own painful battles that at times seemed hopeless. And although I wasn’t brave or a genius, this book gave me hope.

Like your average bacteria after shock, I got my recovery time, and I was *competent*. I do love science, and I could easily get excited about scientific puzzles, creative experiments, and intellectual challenges. I found a true scientific passion, a great postdoc lab, and a vivid and welcoming scientific community. As it turned out, given a healthy body and slightly older kids, I could be a successful scientist after all, and my postdoc years have been one of the most beautiful periods of my life. And then recently, a miraculous thing happened, and I got a faculty position in a wonderful department at a great university. Even better, several students have joined my team, and I’m truly astonished by this fantastic group of hardworking, serious, and motivated young women. Our first New Year’s meeting was dedicated to conversations about goals, strengths, fears, and challenges. The discussion was honest, eye-opening, and on my end, a call to action. “Hey,” I told them, “I have a brilliant idea, we are going to start a book club!” Blank faces staring back, shy smiles covering disbelief. But I was too excited to stop now. “We are going to learn about great scientists who persevered in the face of severe adversity, and I know exactly which book we are going to read!”
